# Investigating the Effect of PCL Concentrations on the Characterization of PLA Polymeric Blends for Biomaterial Applications

**DOI:** 10.3390/ma15207396

**Published:** 2022-10-21

**Authors:** Solechan Solechan, Agus Suprihanto, Susilo Adi Widyanto, Joko Triyono, Deni Fajar Fitriyana, Januar Parlaungan Siregar, Tezara Cionita

**Affiliations:** 1 Department of Mechanical Engineering, Faculty of Engineering, Diponegoro University, Semarang 50275, Indonesia; 2 Department of Mechanical Engineering, Universitas Muhammadiyah Semarang, Kampus Kedungmundu, Semarang 50254, Indonesia; 3 Department of Mechanical Engineering, Sebelas Maret University, Surakarta 57126, Indonesia; 4 Department of Mechanical Engineering, Universitas Negeri Semarang, Kampus Sekaran, Gunungpati, Semarang 50229, Indonesia; 5 Faculty of Mechanical & Automotive Engineering Technology, Universiti Malaysia Pahang, Pekan 26600, Malaysia; 6 Faculty of Engineering and Quantity Surveying, INTI International University, Nilai 71800, Malaysia

**Keywords:** polylactic acid (PLA), polycaprolactone (PCL), ball milling, pressure compaction, sintering

## Abstract

Polylactic acid (PLA) and polycaprolactone (PCL) are synthetic polymers that are extensively used in biomedical applications. However, the PLA/PCL blend produced by ball milling, followed by pressure compaction and sintering, has not been extensively explored. The goal of this research is to investigate the effect of the composition of biomaterials derived from PLA and PCL prepared by ball milling, followed by pressure compaction and sintering, on mechanical and physical properties. PCL and PLA with various concentrations were blended utilizing a ball milling machine for 2 h at an 80-rpm rotation speed. The obtained mixture was placed in a stainless steel 304 mold for the compacting process, which uses a pressure of 30 MPa to create a green body. The sintering procedure was carried out on the green body created at 150 °C for 2 h using a digital oven. The obtained PLA/PCL blend was tested using Fourier transform infrared spectroscopy (FTIR), X-ray diffraction (XRD), Scanning electron microscopy (SEM), density, porosity, and three-point bending. Following the interaction between PCL and PLA in the PLA/PCL blend, the FTIR spectra and XRD diffractograms obtained in this work revealed a number of modifications in the functional groups and crystal phase. The 90PLA specimen had the best mechanical properties, with a maximum force and displacement of 51.13 N and 7.21 mm, respectively. The porosity of the PLA/PCL blend decreased with increasing PLA concentration so that the density and flexural properties of the PLA/PCL blend increased. The higher PCL content decreased the stiffness of the PLA molecular chain, consequently reducing its flexural properties.

## 1. Introduction

When the load on a bone surpasses the inherent strength of the bone, fractures happen. Bone fractures can be repaired by placing fragmented bones in close proximity to one another, preventing them from shifting, and keeping them together. Over time, the bones can mend themselves. External and internal fixation are two methods of bone repair that are frequently used in the field of medicine [1,2].

External fixing entails the application of gypsum, a hard substance wrapped around the outside of the body to prevent displacement of the fractured bone. However, the installation of gypsum can restrict the patient’s mobility. Therefore, internal fixation is a prevalent strategy today. This procedure involves the surgical placement and attachment of a metal plate strengthened with screws to the broken bone. These implants are temporary; after the bones have healed, the plate and screws can be removed surgically [2,3].

In recent times, the research in advanced fields—for instance, medical applications—on biomaterials to replace or recover the tissue functions of the human body or in order to enhance the quality of life has risen substantially [4]. Regenerative medicine and tissue engineering, surgical implants or bone-fixing devices in orthopedic applications, porous structures in tissue engineering, implantable matrices for controlled medication release within the body, as well as absorbable sutures or closures are just a few of the applicable biomaterial applications [4,5]. In both basic research and clinical applications, polymer biomaterials have drawn a lot of attention. Polymers were utilized in the development of biomaterials due to their chemical structures’ adaptability, biocompatibility, biodegradability, and compatibility with required biomolecules [5,6,7]. Polymer biomaterials are classified according to their natural or synthetic origin [7,8,9].

Generally, natural polymers consist of proteins (silk fibroin, collagen) as well as polysaccharides (cellulose, hyaluronic acid, alginate, and chitosan). Biofunctional chemicals present in natural polymers ensure bioactivity, biomimetic surfaces, and natural remodeling [9,10]. However, their significant disadvantages, for instance, insufficient mechanical strength, uncontrollable degradation rate, limited tunability, microbial contamination (for example, endotoxin), as well as immunogenic response, limit their use with respect to bone tissue regeneration [10,11]. On the other hand, the structure and properties of synthetic polymers can be modified by attentively designing the polymer’s functional groups. This advantage guarantees predictable, reproducible, and tunable properties to suit a particular application.

For example, the rate of degradation can be changed by adjusting the chemical composition, crystallinity, and molecular weight. Additionally, synthetic polymers displayed lower osteoconductivity, and cell recognition sites, as well as lower bioactivity when compared with natural polymers. Bioceramics have been tested in this situation to enhance their surface performance with respect to bone tissue regeneration [9,12]. Among the most broadly utilized synthetic polymers are aliphatic polyesters, namely, polylactide (PLA), polycaprolactone (PCL), as well as poly (lactide-co-glycolide) (PLGA) [13,14].

Given their ability to meet a variety of functional requirements for medical devices, including mechanical properties, biodegradability, and biocompatibility, synthetic polymers are increasingly being employed in the medical sector. An additional deciding aspect is the bioactivity or antimicrobial properties when polymers or particle-reinforced coatings are applied [5,9,15,16]. PLA and PCL are extensively utilized synthetic polymers whose properties are being explored for biomedical applications. Further, PLA and PCL also exhibit excellent biocompatibility and biodegradability. In addition, they are non-toxic and degrade at a controlled rate once injected into the human body. 

Compared to PCL, glassy PLA is more brittle and breaks down more quickly, and its flexibility and suppleness are also diminished [17]. Nevertheless, since PCL has low surface energy, there is no attachment signal and less cell adherence to the surface as a consequence [9,18]. In order to create novel biomaterials that go beyond what each polymer can do on its own, these two polymers can be combined effectively [19,20,21].

Regardless of the fact that PCL and PLA are compatible [22,23], research has revealed that when combined using the melt blending process, there is only a relatively faint connection between the two materials [24,25]. According to the research findings by Broz et al. (2003), PCL blends with a PCL concentration of more than 50% showed increased interactions between PLA and PCL [26]. Moreover, Zhai et al. (2009) discovered that the best PCL and PLA mixing was found at the ratio of PLA70/PCL30 [27].

The results of the Dynamic Mechanical Thermal Analysis (DMTA) test conducted by López-Rodriguez et al. (2006) show that there exists a poor interaction between PCL and PLA blends [28]. The impact strength of the PLA/PCL blend increased by around 200% and 350%, respectively, having 30% and 40% PCL [29]. Differential Scanning Calorimetry (DSC), as well as DMTA analysis of the PLA/PCL mixture, showed two Tg (glass transition temperature) values at positions near the raw components, demonstrating a distinct phase separation. This indicates a weak interaction in PLA/PCL blends [22]. It has been reported that PLA and PCL have minimal phase miscibility, as well as numerous coupling agents, which were presented to improve stress transfer from one matrix to another. However, the addition of these compatibilizers has not been seen to make a big difference [5].

The PLA/PCL blend in this research was performed by a ball milling process followed by pressure compaction and sintering. The purpose of this research is to identify the effect of the composition of biomaterials derived from PLA and PCL prepared by ball milling, followed by pressure compaction and sintering, on the physical and mechanical properties. The method in this study is expected to result in a rise in the PLA/PCL blend strength and create a good interface bond. This is required since the method of combining PLA/PCL with ball milling, followed by pressure compaction and sintering, is yet to be researched.

## 2. Materials and Methods

The materials employed in this research were commercial polylactic acid (PLA) and PCL, which had been in the form of powder and pellets, respectively. The PLA utilized was manufactured by Reprapper Tech Co., Kowloon, Hong Kong, with densities, melting temperatures, and grain sizes of 1.24 g/cm^3^, 175–220 °C, and 5–10 µm, respectively [30]. Meanwhile, the PCL material utilized in this study was a small granule manufactured by Solvay Interox Limited, Warrington, UK, with diameters, densities, and melting points of 0.5 mm, 1.1 g/cm^3^, and 58–60 °C, respectively [31]. Fourier transform infrared (FTIR-microscope Spotlight 400, Perkin Elmer, Boston, MA, USA) and X-ray diffraction (Shimadzu XRD-7000 diffractometer, Tokyo, Japan) were used to test PLA and PCL.

**Figure 1:**
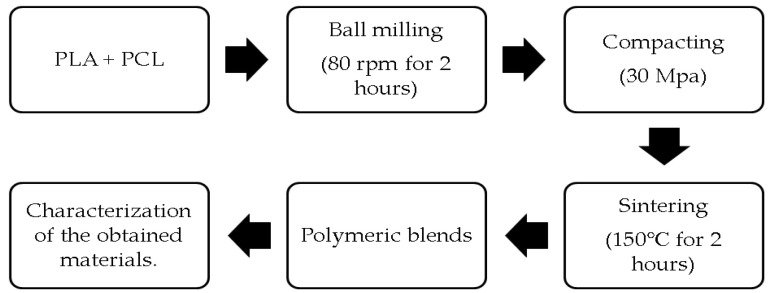
The preparation process of PLA/PCL blend.

The formulation of a PLA/PCL blend is shown in Table 1. The schematic diagram of the procedure for producing a PLA/PCL blend is depicted in Figure 1. PCL and PLA were blended for 2 h in a Laboratory Ball Mill Machine (Bexco; Haryana, India) at an 80-rpm rotation speed. The obtained mixture was placed in a stainless steel 304 mold with a 17-mm diameter and a 3-mm thickness, accordingly. The following stage was the compacting process, which used a pressure of 30 MPa to create a green body. Then, the sintering procedure was carried out on the created green body at 150 °C for 2 h using a digital oven model D1570 made in Taiwan. The obtained PLA/PCL blend was tested using FTIR, XRD, SEM (JSM-6510 Series Scanning Electron Microscope, Tokyo, Japan), density, porosity, and three-point bending.

The Fourier transform infrared (FTIR) method was employed to determine the functional groups composing the material as well as to identify the molecular chain orientation in the PLA/PCL blends [32,33]. The structural changes caused by the processing procedure—for instance, bending or stretching of the functional groups in the polymer—could be examined utilizing FTIR spectra [34]. 

The functional groups that exist in the PLA/PCL blends, PCL and PLA, were determined using FTIR-microscope Spotlight 400 (Boston, MA, USA). Other than that, each spectrum was documented between 400 cm^−1^ and 4000 cm^−1^. Thus, the spectral correction of FTIR spectra was done using Spectrum 10 ES™ software.

X-ray diffraction (XRD) was utilized to examine changes in the crystallinity of the patch, for example, PLA/PCL blend, PCL, and PLA [34]. Additionally, XRD patterns on the PLA/PCL blend, PCL, and PLA were recorded utilizing a Shimadzu XRD-7000 diffractometer (Tokyo, Japan). Scans were performed at 2θ from 10° to 30° at a scanning speed of 2°/min using Ni-filtered CuK*α* radiation at 40 kV as well as 30 mA. A scanning electron microscope (SEM) (JSM-6510 Series Scanning Electron Microscope, Tokyo, Japan) was also employed to observe the materials’ morphology at a 15 kV accelerating voltage [35,36].

The three-point bending test was employed to identify the flexural modulus, flexural strength, and maximum force of the PLA/PCL blend performed under American Society for Testing and Materials (ASTM) D790-17 [37]. Density testing was conducted to identify the PLA/PCL blend density, and the Archimedes method was utilized to calculate the mixture’s actual density based on the ASTM D792-20. As the test medium, water distillate was utilized. The mass of the PLA/PCL blend was identified using an analytical balance. The determination of the actual density, theoretical density, and void volume fraction of the biocomposites was based on studies conducted by Taib et al. (2018) [38] and Satapathy et al. (2017) [39].

## 3. Results and Discussion

**Figure 2:**
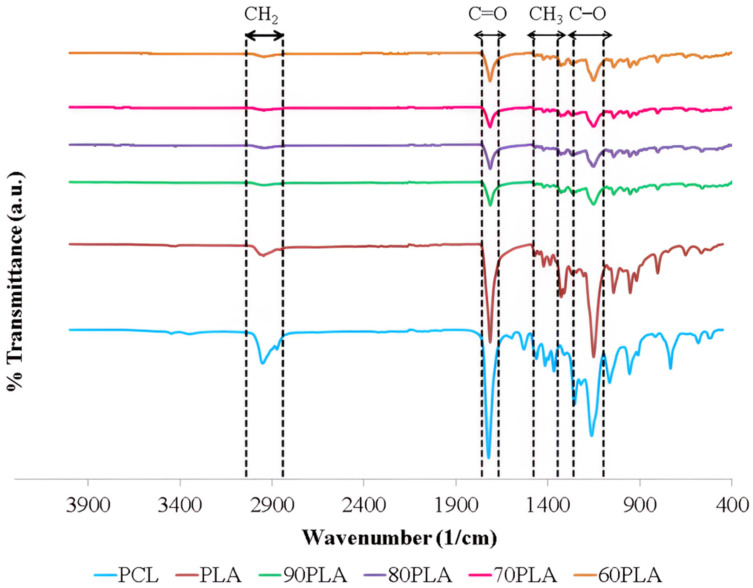
FTIR spectra of samples.

Figure 2 displays the samples’ Fourier transform infrared (FTIR) spectra. Figure 2 demonstrates the FTIR spectrum with changes in chemical structure due to the interaction between the PCL and polylactic acid (PLA) in the PLA/PCL blends. In this research, the acquired PLA/PCL blends formed functional groups with identical wavenumbers. The existence of CH_3_, C=O, as well as C–O peaks in the PLA/PCL blends (90PLA, 80PLA, 70PLA, and 60PLA), indicated the existence of PLA, whereas the presence of C–O, C=O, and CH_2_ peaks reflect PCL content in the PLA/PCL blends (90PLA; 80PLA; 70PLA; as well as 60PLA). A peak at a wavelength of 2915–2940 cm^−1^ implies CH_2_ asymmetric stretch, whereas the peak at 1346–1480 cm^−1^ exhibits CH_3_ symmetric stretch. In addition, the C=O stretch and the C–O stretch are noticed at 1670–1760 cm^−1^ and 1000–1100 cm^−1^, respectively [40,41,42,43]. In the case of the PCL/PLA blend, a spectrum consisting of PCL and PLA was obtained. All these findings clearly indicate that PCL and PLA are present in PCL/PLA mixtures [44]. Per the test results using FTIR, PCL and PLA did not produce new peaks in the spectrum. This demonstrates that PLA and PCL bond mechanically. Alternatively, Chomachayi et al. (2020) and Decol et al. (2018) both achieved identical outcomes obtained from this study. According to their findings, PLA/PCL did not form any new bonds [45,46]. The lack of a significant change in wavenumber shows that the PLA and PCL components did not interact at all when they were blended [47].

The addition of PCL in this study decreased the intensity of bands at C–O, C=O, and CH_3_ in PLA/PCL blends contrasted with pure PLA. The band between 1670–1760 cm^−1^ had less intensity once PCL was added. This occurs because the C=O group in PLA interacts through hydrogen bonding with the –OH group in PCL. This phenomenon was also identified in Sundar et al.’s (2020) investigation [48]. Furthermore, the PLA/PCL blend exhibits the same CH_3_ asymmetric stretching vibrations as pure PLA. The CH_3_ group is pertinent to the PLA material’s hydrophobic nature. The PLA/PCL blend became more hydrophobic because the CH_3_ group did not change [48].

**Figure 3:**
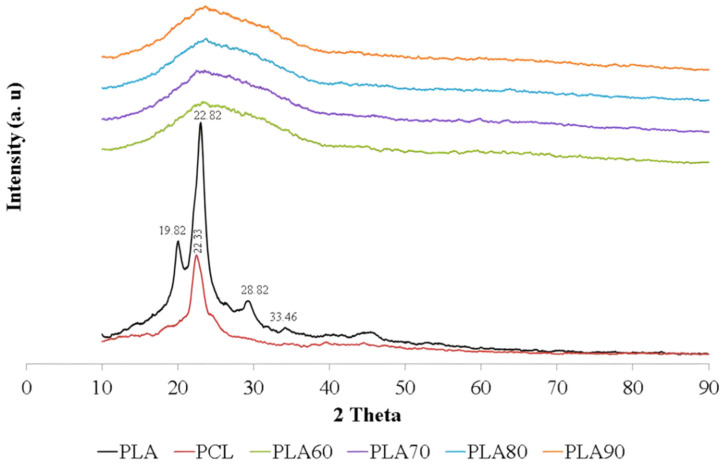
X-ray diffraction (XRD) patterns of samples.

Figure 3 displays the XRD patterns of pure PLA, PCL, and PLA/PCL blends. The X-ray diffraction (XRD) spectra of the PLA/PCL blend were contrasted with those of pure PCL and PLA to locate the characteristic peaks. The three distinct diffusion bands characteristic of the crystalline phase are primarily visible in the diffraction pattern of pure PLA, together with these prominent crystalline peaks at 2θ = 19.66°, 22.82°, and 28.82°. These diffractions could be attributed to the PLA’s α-form crystal, as per the literature. Meanwhile, the PCL pattern has peaks that are roughly at 2θ = 23.33° [49,50]. In this study, the blend of PLA and PCL showed an amorphous nature with a broad hump in all variations of PCL content. The broad hump of the PLA/PCL blend is positioned between 10.00° and 40.00°.

Furthermore, the addition of PCL lowered the percentage of crystallinity in the PLA/PCL blend compared with pure PLA. This might be caused by PLA crystallization interfering with PCL crystallization during cold crystallization. The PLA cold crystallization temperature is roughly 100 °C [51]. Meanwhile, PCL has melted at this stage [23].

The absence of crystal peaks in the PLA/PCL blend indicates that the structure formed is amorphous. This mixture showed an increase in the distance between layers, which was indicated by a broadening of the peak between 2θ of 10.00° and 40.00°. The increase in the distance between the layers of PLA indicated that this mixture had a more amorphous region, whereas the incorporation of PCL resulted in a less regular structure, making crystallization more difficult.

The results of this investigation are consistent with the findings of Silverajah et al. (2012) [52]. Using a melt blend technique, they combined polylactic acid (PLA) and epoxidized palm oil (EPO). The absence of crystal peaks in the mixture of PLA/1 wt% EPO(3) indicated the formation of an amorphous structure. This mixture showed an increase in the distance between the layers, with the distance d increasing from 5.49 Å (pure PLA) to 5.79 Å at the diffraction peak centered at 15.28°. When the distance between PLA layers increased, it showed that this mixture had more amorphous areas than crystalline areas [52]. 

Hou et al. (2019) obtained the XRD pattern of a PLA/PCL mixture with overlapping PCL phase crystal peaks in the amorphous phase of PLA. The large and broad peaks at 2θ located between 10° and 30° show the amorphous zone of PLA [25]. Amorphous PLA in a blend of PLA/PCL, which is characterized by an indistinct peak with a low absorption intensity, was also found in a study conducted by Lu et al. (2016) [53]. This is due to the difference in the degree of deformation in the PLA and PCL molecules during the sample manufacturing (electrospinning) process. The mixed diffraction pattern of PLA/PCL has PLA and PCL peaks with the dominance of the amorphous phase with broad peaks and low absorption intensity. The addition of a low molecular weight PCL polymer to PLA resulted in a sharp decrease in the degree of crystallinity of the PLA/PCL blend and a relatively high amorphous morphology, making it suitable for increasing drug loading efficiency and promoting drug release. In addition, the low level of crystallinity can shorten the degradation time. This can be a solution to the problem of the low degradation rate of the PLA and PCL [53].

All of the PLA/PCL blends developed in this study had smaller diffraction peaks than pure PLA. The filler, which can be either PLA or PCL, has a big effect on the XRD diffraction pattern of the polymer matrix [49]. Sintering carried out on the green body at a temperature of 150 °C for 2 h is responsible for this phenomenon. The most important aspect in influencing the polymers’ mechanical properties is their crystallinity degree. The heat effect, stereoisomer ratio, and production parameters have an effect on the crystallinity of polymers. Here, the crystallinity of polymers is dependent on their thermal history [54].

The findings of this study are identical to those obtained by Thunsiri et al. (2020). According to their findings, the PLA/PCL mixture was also amorphous in nature, with a broad peak ranging between 10° to 25° [55].

Additional research found that the PLA/PCL blend’s crystallization would constantly be lower than that of pure PLA and would worsen as the PCL content rose [49].

Even though it had little influence on the early stages of water transport in the polymer matrix, crystallinity was shown to reduce the pace of PLA degradation. However, it had a substantial effect on the final swelling of specimens and the rate of their biodegradation. Therefore, it could be claimed that the denser structure of the original crystalline material was more resistant to enzyme attachment and oligomer diffusion. Subsequently, this was supported by the observation that the degradation rate rose substantially more quickly and approached that of the amorphous material if the characteristic dimension of the crystalline sample was diminished [54,56].

**Figure 4:**
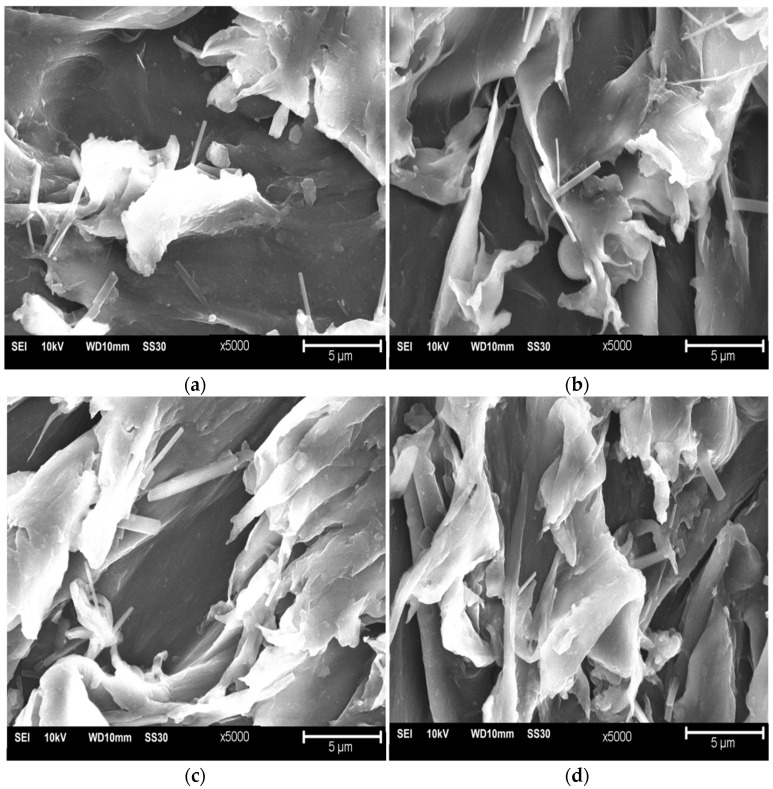
SEM images of surfaces (**a**) 90PLA, (**b**) 80PLA, (**c**) 70PLA, and (**d**) 60PLA at 5000× magnification.

The SEM micrographs of the PLA/PCL blend surfaces consisting of 90PLA, 80PLA, 70PLA, and 60PLA samples are presented in Figure 4. The morphological structure of the white material is PCL, and the black material is PLA [23]. The 90PLA sample in Figure 4a shows less white material than black and is attached to the surface of the PLA material. Meanwhile, the 80PLA sample in Figure 4b for white material is due to the increase in PCL content attached to the PLA surface. The PCL content increases, so the white material is more common in the 70PLA samples, as shown in Figure 4c. The percentages of the white and black colored material are almost the same in the 60PLA sample, where the PCL content is 40 wt% (Figure 4d).

PCL particles dispersed in a matrix of PLA largely formed a bond of PLA/PCL that occurred at the branches and the ends of the white color because they have good adhesive properties [57].

PLA/PCL blends are partially immiscible in the form of small flakes attached to the material’s surface. The morphological structure of phase separation, given the immiscibility between PCL and PLA, is evident in the vastly different melting temperatures [58]. Although some flakes are immiscible and some voids are caused by their detaching, this indicates that PCL can improve PLA compatibility. The increase in PCL content resulted in a rougher surface with many voids and flake structures in the 80PLA, 70PLA, and 60PLA specimens. This demonstrates that when PCL concentration rises, plastic deformation also rises [59].

**Figure 5:**
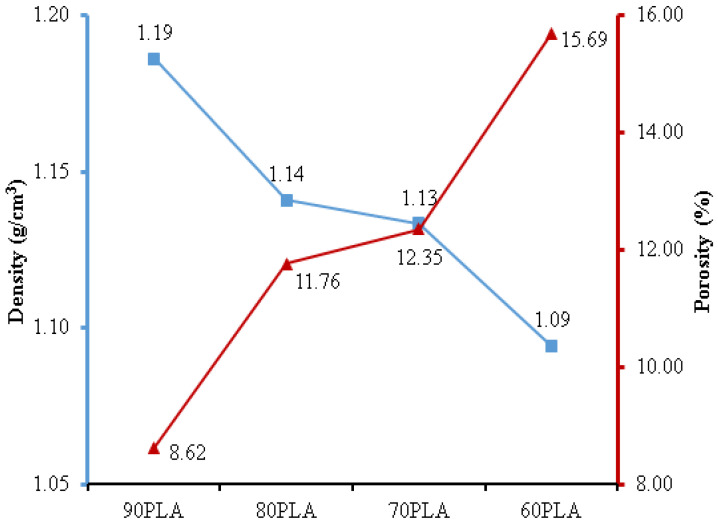
The density and porosity of PLA/PCL blends.

The density test results depict that the 90PLA sample had a maximum density of 1.19 g/cm^3^ (Figure 5). Conversely, the rise in PCL content in 80PLA, 70PLA, and 60PLA samples resulted in a decrease in density of 1.14 g/cm^3^, 1.13 g/cm^3^, and 1.09 g/cm^3^, respectively. Since PCL has a lower density than PLA, the density of the PLA/PCL blend decreased. Furthermore, PCL has a density of 1.1 g/cm^3^, whereas PLA has a density of 1.24 g/cm^3^ [60]. The lowest porosity was found in the 90PLA sample, with a porosity of 8.62%, and the highest porosity was found in the 60PLA sample, at 15.69%. The density of the PLA/PCL blend was fully affected by the porosity value, and the density decreased with the increase in porosity [61]. According to the findings of this study, the density of PLA/PCL blends increased as their PLA content increased. This study resulted in a PLA/PCL blend with densities (90PLA, 80PLA, and 70 PLA) that matched the density values in the human cortical bone, which denotes 1.1–1.3 g/cm^3^ [37].

The findings of this research are consistent with those of Qiao et al.’s (2021) investigation, which found that when the PLA content was raised, the density of the PLA/PCL blend also increased [62]. The mixing ratio had a significant influence on the values of bulk density, apparent density, and porosity. The porosity of the PLA/PCL blends decreased as the PLA concentration increased. Additionally, the bulk density and apparent density of PLA/PCL blends increased linearly as PLA content increased [63]. Zihui et al. (2020) produced a variety of melt-blended PCL/PLA blends with distinct dispersed phase morphologies dependent on the PCL matrix. As PLA content increased, pore size decreased, pore density increased, pore distribution became more uniform, and apparent density increased [64].

**Figure 6:**
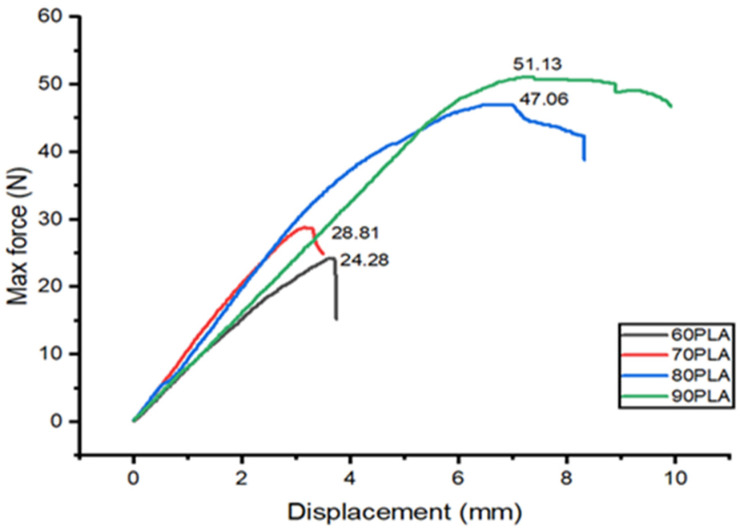
The flexural test results on PLA/PCL blend.

The results of the three-point bending test on the PLA/PCL blend are shown in Figure 6. The highest flexural strength is produced at 90PLA with a maximum force and displacement of 51.13 N and 7.21 mm, respectively. The increase in PCL content caused a decrease in flexural strength and displacement in the PLA/PCL blend. The lowest flexural strength and displacement were found in the 60PLA specimen, with a maximum force and displacement of 28.81 N and 3.28 mm, respectively. The greater the PLA composition, the higher the mechanical strength [19,65]. The increase in PCL content resulted in decreased flexural strength because PCL had low mechanical properties [57]. In addition, the ability to absorb energy would decrease with increasing PCL composition so that the flexural strength would also decrease. These findings aligned with the research by Ferri et al. (2016), stating that the specimen’s flexural strength would decrease with increasing PCL content [22].

The good interfacial adhesion between PCL and PLA in the 90PLA specimens led to an increase in maximum stress and flexural strength. The increase in PCL content in the 80PLA, 70PLA, and 60PLA specimens caused the flexural strength to decrease because the dispersion state of PCL in the PLA matrix deteriorated at the high PCL content. Furthermore, the addition of 10 wt.% PCL to the 90PLA specimens can improve the stress transfer and allow the formation of a stiffer bond, thereby contributing to the improvement of the flexural properties of the PLA/PCL blends. The high PCL content can reduce the stiffness of the PLA molecular chains, causing a decrease in flexural properties. Increasing the PCL content above 10 wt.% can increase the occurrence of cavities in the mixture. This causes a stress riser to form in the PLA/PCL mix, which causes the flexural strength to decrease. 

Chen et al. (2018) revealed that the flexural strength of the mixture increased with increasing PCL content up to 2.5 wt.%. This is due to the good interfacial adhesion between the PCL and PLA matrices in the PLA/PCL mixture. If the amount of PCL in the mixture increases by more than 2.5 wt%, the flexural strength of the mixture decreases because the PCL is less evenly distributed in the PLA matrix [66]. The findings in this study are in accordance with research conducted by Silverajah et al. (2012). Their research demonstrated that the inclusion of 1 wt.% EPO in a PLA/EPO blend permitted the creation of stronger bonds, which contributed to the improvement of flexural properties. Flexural strength and modulus decrease of 43% and 13%, respectively, with 5% EPO loading [52].

## 4. Conclusions

The PLA/PCL blend has been successfully made using ball milling, followed by pressure compaction and sintering. The morphology, physical properties, and mechanical properties of the obtained PLA/PCL blends were examined using multiple techniques. The results of this study show that the amount of PCL had a huge effect on the characterization of the PLA/PCL blend. The incorporation of PCL in this study decreased the intensity of bands at C–O, C=O, and CH_3_ in PLA/PCL blends as contrasted with pure PLA relying on the Fourier transform infrared (FTIR) spectrum. In the case of the PCL/PLA blend, a spectrum consisting of PCL and PLA was obtained. All these findings clearly indicate that PCL and PLA were present in PCL/PLA mixtures. The lack of a significant change in wavenumber shows that the PLA and PCL components did not interact at all when they were blended.

Furthermore, the absence of crystal peaks in the PLA/PCL blend indicates that the structure formed was amorphous. This mixture showed an increase in the distance between layers, which was indicated by a broadening of the peak between 2θ of 10.00° and 40.00°. The increase in the distance between the layers of PLA indicated that this mixture had a more amorphous region, whereas the incorporation of PCL resulted in a less regular structure, making crystallization more difficult. The increase in PCL content resulted in a rougher surface with many voids and flake structures in the 90PLA, 80PLA, 70PLA, and 60PLA specimens. This proves that plastic deformation increased with the rise in PCL content. The mixing ratio had a significant influence on the values of bulk density, apparent density, and porosity. The porosity of the PLA/PCL blends decreased as the PLA concentration increased.

Additionally, the density of PLA/PCL blends increased linearly as PLA content increased. The 90PLA sample had the highest density (1.19 g/cm^3^) and the least amount of porosity (8.62%). The flexural strength of the PLA/PCL blend increased as its density increased. The 90PLA sample had the best mechanical properties, with a maximum force and displacement of 51.13 N and 7.21 mm, respectively. The addition of 10 wt.% PCL to the 90PLA sample can improve the stress transfer and allow the formation of a stiffer bond, thereby contributing to the improvement of the flexural properties of the PLA/PCL blends. Furthermore, the high PCL content can reduce the stiffness of the PLA molecular chains, causing a decrease in flexural properties.

## Figures and Tables

**Table 1:** Labels and composition of samples studied.

Sample Codes	PLA (wt.%)	PCL (wt.%)
90PLA	90	10
80PLA	80	20
70PLA	70	30
60PLA	60	40

## Data Availability

Data are contained within the article.
